# Different patterns of leukocyte immune responses to infection of ancestral SARS-CoV-2 and its variants

**DOI:** 10.3389/fcimb.2025.1508120

**Published:** 2025-04-17

**Authors:** Yuanyuan Wu, Raphael Serna, Wenqi Gan, Zhichao Fan

**Affiliations:** ^1^ Department of Immunology, University of Connecticut School of Medicine, Farmington, CT, United States; ^2^ Department of Public Health Sciences, University of Connecticut School of Medicine, Farmington, CT, United States

**Keywords:** COVID-19, SARS-CoV-2 variant, leukocyte, neutrophil, monocyte, lymphocyte

## Abstract

**Background:**

Contributions of leukocytes to severe acute respiratory syndrome coronavirus 2 (SARS-CoV-2) defense have been reported extensively. However, it remains unclear whether there are different leukocyte responses to ancestral SARS-CoV-2 and its variants.

**Methods:**

We analyzed peripheral blood leukocyte and subtype concentrations from 575 COVID-19 patients and 950 non-COVID-19 subjects registered at the University of Connecticut John Dempsey Hospital between 2020 and 2022, which covers the ancestral strain, Delta, and Omicron variants.

**Results:**

We found that neutrophils, immature granulocytes, and monocytes were elevated, and lymphocytes were reduced after infection. These hyperactive neutrophils/immature granulocytes and suppressed lymphocytes/monocytes were associated with poorer prognosis in ancestral strain infection. Different from the ancestral strain, hyperactive immature granulocytes were not shown in the decedents of Delta infection, and immature granulocyte concentration was not observed to be associated with mortality. In Omicron infection, suppressed lymphocytes and monocytes were not shown in the decedents, and lymphocyte/monocyte concentrations were not associated with mortality.

**Conclusions:**

Our findings provided insights into different leukocyte immune responses to ancestral SARS-CoV-2, Delta, and Omicron variants.

## Introduction

Since the first report of novel severe acute respiratory syndrome coronavirus 2 (SARS-CoV-2) in December 2019 (Zhang et al., 2020), the coronavirus disease 2019 (COVID-19) pandemic has caused more than 769 million cases and 6.9 million deaths worldwide, according to the World Health Organization (WHO) dashboard (accessed 30/08/2023). The heterogeneous disease courses of COVID-19 range from mild to severe and even fatal outcomes (Hu et al., 2021). To better prevent and control COVID-19, researchers put huge effort into identifying clinical characteristics and infectious mechanisms. Numerous variants have occurred worldwide because of the high genetic recombination and mutation of coronaviruses (Aleem et al., 2023). Five dominant variants were defined by the WHO, including Alpha (B.1.1.7), Beta (B.1.351), Gamma (P.1), Delta (B.1.617.2), and Omicron (B.1.1.529) (Aleem et al., 2023). The Delta variant predominantly occurred from May 1, 2021, and was quickly overtaken by the Omicron variant in December 2021 (Lambrou et al., 2022). Although the Omicron variant presented an increased transmission compared to previous variants (Kumar et al., 2022), it is reported that Omicron has less pathogenic potential, which may partly explain the reduced morbidity of COVID-19 in 2022 (Zhang HP. et al., 2023). Thus, the SARS-CoV-2 variants may have different pathogenesis compared with the ancestral strain.

The COVID-19 pathology results from not only the viral infection but also abnormal immune responses. Previous studies have shown that blood leukocyte concentrations are associated with COVID-19 progression and prognosis, such as substantially higher concentrations of neutrophils (Kasine et al., 2022) and immature neutrophils (Obermayer et al., 2021), highly dysregulated monocytes (Schulte-Schrepping et al., 2020), and a lower concentration of lymphocytes (Zhang et al., 2021). However, it remains unclear whether the ancestral SARS-CoV-2 and its variants have different effects on the immune system, resulting in differences in peripheral blood leukocytes. Therefore, we analyzed the 2020–2022 hematology test data from the University of Connecticut (UConn) John Dempsey Hospital to examine the associations between leukocytes and leukocyte subtypes and the morbidity and mortality of different types of SARS-CoV-2 infection. Our investigation shed light on the tendency of immunity to vary; this information is a precondition for identifying diagnostic markers and therapeutic strategies targeting COVID-19 in the future.

## Materials and methods

### Study population and data collection

The unidentified participants were randomly enrolled from the electronic databases of the UConn John Dempsey Hospital in Farmington, Connecticut, from 2020 to 2022. Here, we leverage the hospital database to build a clinical study on patients infected with COVID-19 from March 2020 to December 2022, as well as a group of non-COVID-19 controls who were annual physical examination patients with no symptoms of SARS-CoV-2 infection within 2 weeks before the examination. Please note that it is possible that there could be asymptomatic COVID-19 individuals in the group of non-COVID-19 controls. An automated Sysmex XN-3000 analyzer was used to measure the peripheral blood white blood cell (leukocyte) counts.

**Figure 1:**
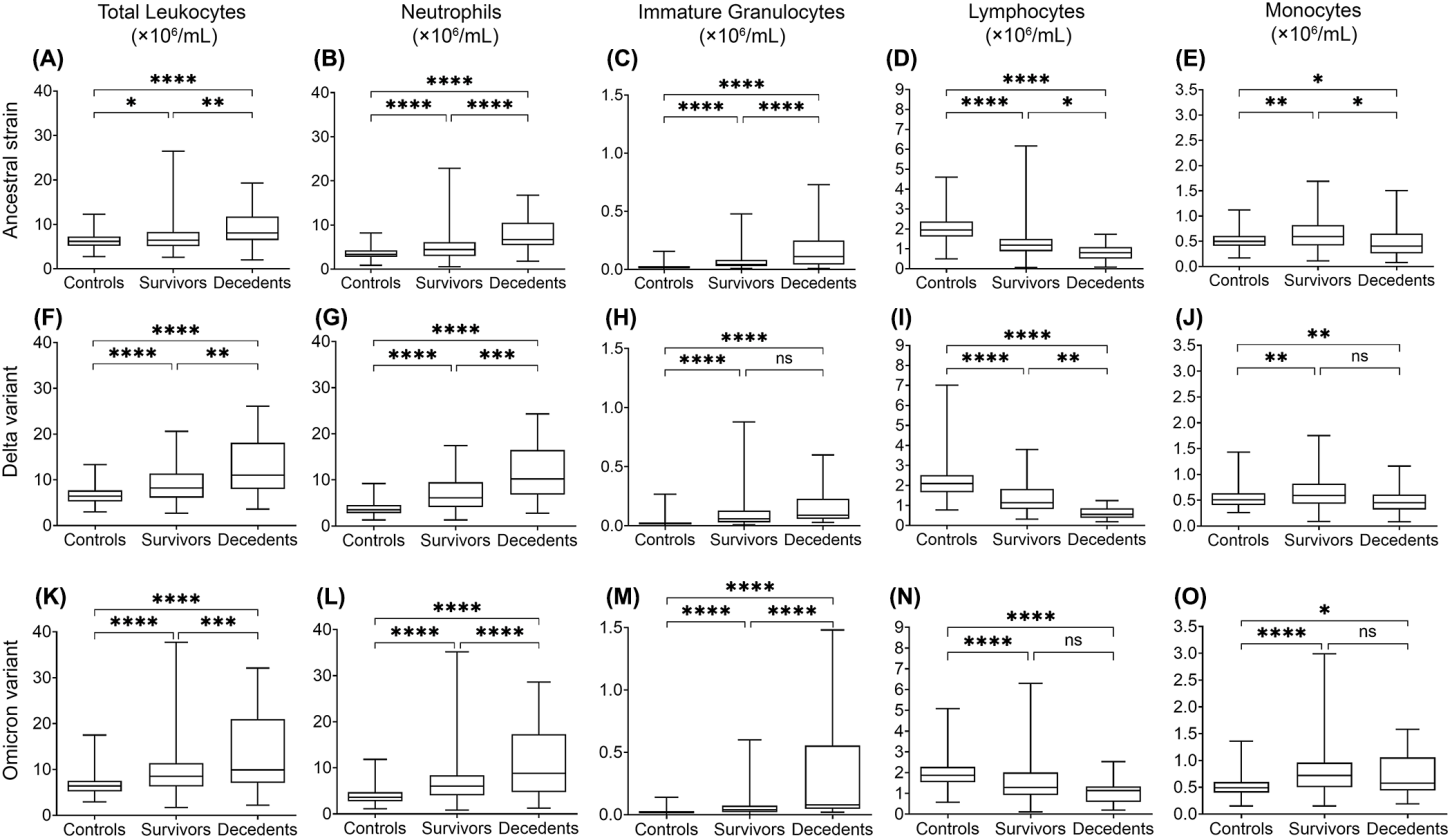
Multiple linear regression analysis of leukocyte levels in COVID-19 survivors and decedents compared to non-COVID-19 control subjects for 2020 (mainly ancestral strain), 2021 (mainly Delta variant), and 2022 (mainly Omicron variant). Total leukocytes **(A, F, K)**, neutrophils **(B, G, L)**, immature granulocytes **(C, H, M)**, lymphocytes **(D, I, N)**, and monocytes **(E, J, O)** indicate changes of significant difference in 2020 **(A-E)**, 2021 **(F-J)** and 2022 **(K-O)**. Data are presented as mean ± SD. ns, P>0.05, *P < 0.05, **P < 0.01, ***P < 0.001, ****P < 0.0001.

**Figure 2:**
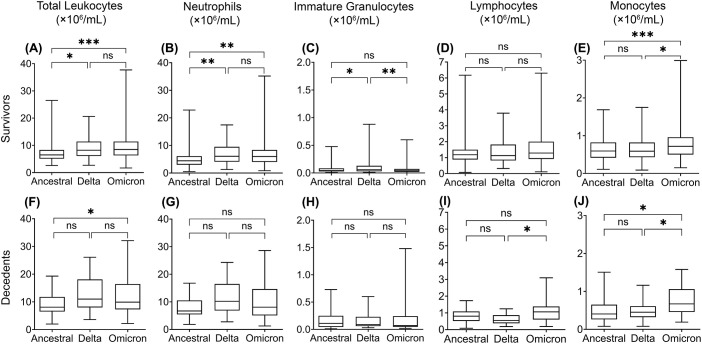
Multiple linear regression analysis of leukocyte levels in COVID-19 survivors (upper) and decedents (lower) infected with the ancestral strain, Delta variant, and Omicron variant. Total leukocytes **(A, F)**, neutrophils **(B, G)**, immature granulocytes **(C, H)**, lymphocytes **(D, I)**, and monocytes **(E, J)**. Data are presented as mean ± SD. ns, P>0.05, *P < 0.05, **P < 0.01, ***P < 0.001, ****P < 0.0001.

Patient demographics, including survival, age, sex, COVID-19 vaccination status, corticosteroid use, and comorbid conditions, were recorded during hospital admissions and summarized in **Table 1**. Comorbidities include chronic lung diseases, cardiovascular diseases, diabetes, and cancer. Clinical hematology testing was performed one or several times after hospital admissions, and we summarized patients’ first-time results of leukocyte and subtype counts in peripheral blood (**Figures 1**, **2**). The concentrations of neutrophils, immature granulocytes, lymphocytes, and monocytes were calculated based on the total leukocyte counts (leukocyte count × percentage of subtype/100). According to the Centers for Disease Control and Prevention (CDC) genomic surveillance for SARS-CoV-2 variants (Lambrou et al., 2022), we approximately divided the patients into three groups: 1) ancestral strain infection from March to June 2020; 2) Delta variant infection from July to December 2021; and 3) Omicron variant infection from January to December 2022.

**Table 1:** Demographic characteristics of participants ^a^.

Characteristic	Ancestral strain (2020 March-June)				Delta variant (2021 July-December)				Omicron variant (2022 January-December)			
	Controls (n=350)	Patients (n=186)		*P*-value	Controls (n=300)	Patients (n=94)		*P*-value	Controls (n=300)	Patients (n=295)		*P*-value
		Survivors	Decedents			Survivors	Decedents			Survivors	Decedents	
		(n =159)	(n = 27)			(n = 79)	(n = 15)			(n = 266)	(n = 29)	
Age (years)	49 ± 16	63 ± 15	75 ± 14	<0.001	47 ± 16	58 ± 20	73 ± 15	<0.001	49 ± 16	65 ± 21	73 ± 18	<0.001
Sex (%)				<0.001				0.272				0.024
Male	130 (37.1)	110(69.2)	21 (77.8)		125 (41.7)	40 (50.6)	8 (53.3)		123 (41.0)	126 (47.4)	19 (65.5)	
Female	220 (62.9)	49(30.8)	6 (22.2)		175 (58.3)	39 (49.4)	7 (46.7)		177 (59.0)	140 (52.6)	10 (34.5)	
Vaccination (%)				0.766				<0.001				<0.001
No	349 (99.7)	159 (100)	27 (100)		81 (27)	57 (72.2)	10 (66.7)		43 (14.3)	75 (28.2)	10 (34.5)	
Yes	1 (0.3)	0 (0)	0 (0)		219 (73.0)	22 (27.8)	5 (33.3)		257 (85.7)	191 (71.8)	19 (65.5)	
Corticosteroid use (%)				0.351				<0.001				<0.001
No	224 (64)	109 (68.6)	15 (55.6)		205 (68.3)	10 (12.7)	1 (6.7)		193 (64.3)	87 (32.7)	7 (24.1)	
Yes	126 (36)	50 (31.4)	12 (44.4)		95 (31.7)	69 (87.3)	14 (93.3)		107 (35.7)	179 (67.3)	22 (75.9)	
Comorbidity ^b^ (%)				0.003				0.161				<0.001
No	254 (72.6)	93 (58.5)	15 (55.6)		218 (72.7)	61 (77.2)	8 (53.3)		230 (76.7)	142 (53.4)	12 (41.4)	
Yes	96 (27.4)	66 (41.5)	12 (44.4)		82 (27.3)	18 (22.8)	7 (46.7)		70 (23.3)	124 (46.6)	17 (58.6)	

a Data are presented as mean ± SD for age and number of participants (column percentage) for categorical variables.

b Data indicate any comorbidity, including chronic lung diseases, cardiovascular diseases, diabetes, and cancer.

### Statistical analysis

To further understand the physiological and immunopathological differences among the ancestral SARS-CoV-2 strain, the Delta variant, and the Omicron variant, we analyzed the 2020, 2021, and 2022 data separately. The COVID-19 patients were divided into two groups - COVID-19 survivors and decedents. Sex, age, COVID-19 vaccination status, corticosteroid usage, and comorbid conditions were compared statistically between non-COVID-19 controls and COVID-19 patients each year. The two-sample t-test was used to compare continuous variables. The chi-square test was used to compare categorical variables. Multiple linear regression analysis was performed to examine the relationships between leukocytes and their subtypes with COVID-19 disease outcomes during the three years following adjustment for age, sex, COVID-19 vaccination, corticosteroid use, and comorbid conditions.

Multiple logistic regression analysis was conducted to examine the associations of mortality with leukocytes and each subtype after adjustment for sex, age, COVID-19 vaccination status, corticosteroid use, and comorbid conditions. Adjusted odds ratios (ORs) and 95% confidence intervals (CIs) were calculated by exponentiating the regression coefficients of each cell type. Based on the multiple logistic regression model, we calculated and plotted the predicted mortality probabilities and corresponding 95% CIs. Specifically, for each cell type, we generated a sequence of cell values across the observed range and constructed a new dataset comprising all possible combinations of these cell values, categorical covariates, and mean age. Predicted probabilities were then obtained for each combination using the prediction function in the logistic regression model. For each cell value, we calculated the mean predicted mortality probability and derived the 95% CI using the quantile function. All statistical tests were 2-sided, and a P-value less than 0.05 was considered statistically significant. All statistical analyses were performed using GraphPad Prism version 9.0.0 and R version 4.4.1.

## Results

This study included 575 COVID-19 patients and 950 non-COVID-19 patient controls (**Table 1**). Based on the published CDC results of SARS-CoV-2 variant changes (Lambrou et al., 2022), we divided the patients into three groups: 1) The ancestral strain: 186 patients from March to June 2020, which were mainly ancestral strain infection; 2) The Delta variant: 94 patients from July to December 2021, which were mainly Delta variant infection; and 3) The Omicron variant: 295 patients from January to December 2022, which were mainly Omicron variant infection. The mortality rates for each of the three groups were 14.5% (27 decedents), 16.0% (15 decedents), and 9.8% (29 decedents), respectively.

### Age and sex are correlated with COVID-19

Compared with the non-COVID-19 controls, the average age was significantly higher for the COVID-19 survivors and decedents (**Table 1**), showing that patients hospitalized for COVID-19 are older than patients hospitalized for other issues. This is consistent with the previous studies showing that older people, especially those with comorbidities, might be more likely to be infected and have poorer prognosis (Attaway et al., 2021).

Compared with the non-COVID-19 controls, the survivors and decedents were more likely to be male. However, the sex difference was non-significant for the Delta variant group due to the small sample sizes (**Table 1**). The results are consistent with previous studies (Garg et al., 2020; Shrock et al., 2020; Takahashi et al., 2020; Webb Hooper et al., 2020) showing that males might be more vulnerable to COVID-19 than females. This may be because of differences in ACE2 expression (Peng et al., 2021; Viveiros et al., 2022), hormonal influences (Selek et al., 2021; Sciarra et al., 2023), immune response variations (Klein and Flanagan, 2016; Takahashi and Iwasaki, 2021; Zhao et al., 2021), and other biological or behavioral factors (Friske and Spanagel, 2023).

### COVID-19 vaccination, corticosteroid use, and comorbid conditions

Vaccination status correlates with COVID-19, where COVID-19 patients have a significantly lower vaccination rate than non-COVID-19 controls (**Table 1**). Corticosteroid is commonly used in several respiratory diseases, including COVID-19. As expected, COVID-19 patients have a much higher percentage of corticosteroid use than non-COVID-19 controls (**Table 1**). There is also a higher percentage of COVID-19 patients with comorbidities than non-COVID-19 controls (**Table 1**), suggesting a correlation between COVID-19 and other diseases.

### Higher concentrations of total leukocytes and neutrophils in COVID-19

To assess whether leukocyte responses differ between infection of ancestral SARS-CoV-2 and variants, we analyzed the concentrations of peripheral blood leukocytes and subtypes from COVID-19 patients and non-COVID-19 controls from various aspects. We performed multiple linear regression analyses to compensate for the effects of age, sex, COVID-19 vaccination, corticosteroid use, and comorbid conditions. We then compared the average leukocyte and subtype concentrations between COVID-19 survivors, decedents, and non-COVID-19 controls (**Figure 1**), where the ancestral strain, Delta, and Omicron variant groups were analyzed separately. In addition, we compared leukocyte differences between the three variant groups (**Figure 2**) in survivors or decedents. We then performed multiple logistic regression for each group separately to determine whether leukocytes or subtypes are associated with COVID-19 mortality (**Table 2**).

**Table 2:** Adjusted ORs (95% CIs) for COVID-19 mortality in relation to the concentrations of leukocytes and the subtypes, stratified by three types of COVID-19 infection.

Year	Leukocyte and subtypes	Adjusted OR^a^	95% CI		*p* value
			Lower	Upper	
Ancestral strain (March – June, 2020)^b^	Leukocytes	1.16	1.04	1.30	0.010
	Neutrophils	1.25	1.09	1.43	0.001
	Monocytes	0.20	0.04	1.05	0.057
	Lymphocytes	0.16	0.05	0.54	0.003
	Immature Granulocytes^c^	2.28	1.36	3.82	0.002
Delta variant (July – December, 2021)	Leukocytes	1.21	1.06	1.39	0.006
	Neutrophils	1.27	1.09	1.49	0.002
	Monocytes	0.08	0.00	1.41	0.084
	Lymphocytes	0.04	0.00	0.42	0.007
	Immature Granulocytes^c^	1.59	0.86	2.92	0.137
Omicron variant (January – December, 2022)	Leukocytes	1.12	1.05	1.20	<0.001
	Neutrophils	1.14	1.06	1.23	<0.001
	Monocytes	0.68	0.23	2.04	0.494
	Lymphocytes	0.69	0.38	1.23	0.204
	Immature Granulocytes^c^	2.51	1.65	3.80	<0.001

OR, odds ratio; CI, 95% confidence interval.

a Adjusted ORs (95% CIs) were derived from multiple logistic regression analyses after adjustment for age, sex, comorbidity, corticosteroid use, and COVID-19 vaccination status when appropriate.

b Adjusted for age, sex, comorbidity, and corticosteroid use. No patients in the ancestral strain group received COVID-19 vaccination.

c The original data were right-skewed. Thus, log-transformed data were used in the analysis.

We found that in all three groups, total leukocyte and neutrophil concentrations were significantly higher in COVID-19 survivors and further higher in COVID-19 decedents compared with the non-COVID-19 controls (**Figures 1A, F, K**). The higher leukocyte concentration is mainly contributed by the higher neutrophil concentration (**Figures 1B, G, L**) because neutrophils are the most abundant leukocytes in human blood. Multiple logistic regression analyses showed that total leukocytes and neutrophils were positively associated with COVID-19 mortality (**Table 2**, **Supplementary Figure 1A–B, F-G, K-L**). These imply that the elevation of neutrophil levels may be correlated with COVID-19 pathogenesis and prognosis.

When comparing the three groups, we found that the Delta and Omicron variant survivors had significantly higher total leukocyte and neutrophil concentrations than the ancestral strain survivors (**Figures 2A,B**). Moreover, Omicron variant survivors also have significantly higher monocyte concentrations than the ancestral strain survivors (**Figure 2E**). Omicron but not Delta variant decedents showed significantly higher total leukocyte concentrations than the ancestral strain decedents (**Figure 2F**). This is due to the higher monocyte (**Figure 2J**) but not neutrophil concentration (**Figure 2G**).

### The concentration of immature granulocytes is higher in COVID-19

The increase of immature granulocytes in peripheral blood happens in many inflammatory diseases, suggesting emergency granulopoiesis and cytokine storm (Polidoro et al., 2020; Combadiere et al., 2021; Malengier-Devlies et al., 2021). As expected, COVID-19 also showed a higher concentration of immature granulocytes in peripheral blood (**Figures 1C, H, M**). The COVID-19 decedents showed significantly higher peripheral blood immature granulocytes than survivors during the ancestral strain and Omicron variant infection (**Figures 1C, M**) but not in the Delta variant infection (**Figure 1H**). Consistent with this result, multiple logistic regression analyses showed that immature granulocyte concentrations are positively associated with COVID-19 mortality in the ancestral strain and Omicron variant infection but not the Delta variant infection (**Table 2**; **Supplementary Figure 1**). When comparing the three groups, we found that the Delta variant survivors had significantly higher immature granulocyte concentrations than the other two groups (**Figure 2C**), suggesting that the Delta variant may induce more emergency granulopoiesis. The decedents showed no difference in immature granulocyte concentrations between groups (**Figure 2H**).

### A lower lymphocyte concentration in COVID-19

Compared with the non-COVID-19 controls, the COVID-19 patients had fewer lymphocytes in all three groups, especially for patients who died from COVID-19 (**Figures 1D, I, N**). The results are consistent with the previous studies (Huang et al., 2021; Zhang et al., 2021; Belaid et al., 2022). However, the Omicron variant decedents did not show a further lower concentration of lymphocytes compared to survivors (**Figure 1N**). In addition, lymphocyte concentrations were negatively associated with COVID-19 mortality, although the association was non-significant for those infected with the Omicron variant (**Table 2**; **Supplementary Figure 1D, I, N**), suggesting suppression of regulatory immunity may contribute to a worse prognosis in the ancestral strain and Delta variant but not Omicron variant-caused COVID-19. This was further supported by the results that the Omicron variant decedents (**Figure 2I**) had higher lymphocyte concentrations than those in the Delta variant decedents. The suppression of regulatory immunity might be due to the administration of corticosteroids to the patients.

### Loss of monocytes is associated with poor prognosis in the ancestral strain and Delta variant infection

We found that COVID-19 survivors had significantly higher monocyte concentrations than non-COVID-19 controls (**Figures 1E, J, O**). However, the situation was different for the decedents. Compared with the non-COVID-19 controls, the monocyte concentration in the decedents was significantly higher in the Omicron variant infection (**Figure 1O**) but significantly lower in the ancestral strain infection (**Figure 1E**) and the Delta variant infection (**Figure 1J**). Additionally, monocyte concentrations were negatively associated with COVID-19 mortality, although the association was less significant in the Omicron variant infection (**Table 2**; **Supplementary Figure 1**), suggesting the failed induction of monocyte immunity might be involved in the prognosis of the ancestral SARS-CoV-2 and Delta variant but not Omicron variant caused COVID-19. By comparing the groups, we found the Omicron variant survivors (**Figure 2E**) and decedents (**Figure 2J**) had significantly higher monocyte concentrations than those in the ancestral strain and Delta variant infection, implying a possibility that the Omicron variant may have less capacity to suppress monocyte immunity.

## Discussion

The ancestral SARS-CoV-2 strain has a high pathogenicity, lethality, and genetic variability (Schwab et al., 2023). In the past three years, many variants have been identified. Their lethality seems decreased but exhibits higher transmissibility, including Delta and Omicron variants (Liu and Rocklov, 2021; Chavda et al., 2022). Investigating human immune regulation in response to SARS-CoV-2 variant infection is crucial to understanding further how the elevated inflammatory signaling has contributed to COVID-19 morbidity and mortality. Many studies have focused on the dysregulation of leukocyte response and consequent immunopathology triggered by SARS-CoV-2 (Jimenez and Torres Arias, 2022; Islam et al., 2023). Neutrophilia, hyperactive immature granulocytes, and lymphopenia were usually seen in severe COVID-19 patients (Huang et al., 2020; Tan et al., 2020; Aschenbrenner et al., 2021; Sinha et al., 2022; Almendro-Vazquez et al., 2023; Nasrollahi et al., 2023; Rice et al., 2023). They may cause an excessive inflammatory response to SARS-CoV-2 (Polidoro et al., 2020; Qin et al., 2020; Root-Bernstein, 2023), which is thought to be the main contributor to the death of COVID-19 patients (Mehta et al., 2020). However, no clear illumination exists about the comparison among leukocyte subtypes responding to ancestral strain and its variants during the last three years. Our study compared the distinct leukocyte responses to ancestral SARS-CoV-2, Delta, and Omicron in COVID-19 survivors and decedents.

We were able to show different peripheral blood leukocyte subtype patterns between the ancestral strain, Delta variant, and Omicron variant infection, which may help us understand the difference in immune responses between the SARS-CoV-2 variants. Although COVID-19 patients in all years showed higher concentrations of myeloid cell populations (neutrophils, immature granulocytes, and monocytes) and lower concentrations of adaptive immune lymphocytes compared to non-COVID-19 controls, COVID-19 survivors and decedents show different patterns. In the ancestral strain infection, a significantly higher concentration of neutrophils and immature granulocytes and a lower concentration of lymphocytes and monocytes were shown in decedents compared to survivors, suggesting hyperactivation of neutrophils/granulocytes and suppression of lymphocytes/monocytes may contribute to mortality in ancestral COVID-19. In the Delta variant infection, compared to the ancestral strain infection, a higher concentration of immature granulocytes was not significantly observed in decedents compared to survivors. This is because the Delta variant survivors already induced a high number of immature granulocytes. In the Omicron variant infection, unlike the ancestral strain infection, lower concentrations of lymphocytes and monocytes were not significantly observed in decedents compared to survivors. Actually, Omicron decedents had higher amounts of lymphocytes and monocytes compared to those in the ancestral strain and Delta variant infection. These imply that the mortality observed from the Omicron variant infection might not be due to the inhibition of lymphocytes and monocytes. Detail mechanisms remain for further investigation.

One important finding is the involvement and changes of monocytes during infection of the ancestral strain and different variants. Studies have indicated that the monocyte population is a trigger and target in the progression of severe COVID-19 disease (Schulte-Schrepping et al., 2020; Ahern et al., 2022; Zhang PP. et al., 2023). Studies showed classic monocytes reduced in COVID-19 cases, while increased activated monocyte subsets may impact pulmonary function in COVID-19 convalescents (Payen et al., 2020; Park et al., 2023). However, these studies did not address whether monocyte responses differ in patients infected with ancestral SARS-CoV-2 strain and variants; here, we provide some insights into this question and suggest further investigation. According to these findings, blood monocyte tests could represent a promising routine tool for monitoring the progression of SARS-CoV-2 infection, clinical prognosis of COVID-19, and therapeutic targeting throughout hospitalization.

One pitfall of our research is that we assume patients from 2020 March-June as the ancestral strain infection, patients from 2021 July-December as the Delta variant infection, and patients from 2022 January-December as the Omicron variant infection based on previous published CDC results (Lambrou et al., 2022). This is due to the feasibility limitation of sequencing on all our patients, and this is the best way we can analyze existing patient data and discover differences between ancestral SARS-CoV-2 and its variants. Meanwhile, although we considered COVID-19 vaccination, corticosteroid use, and comorbidities, we did not have data on lifestyle risk factors and genetic factors, and we could not control for the influences of these factors. The number of decedents in each type of infection is small, which may partly explain the non-significant associations for decedents.

## Conclusion

Collectively, our study demonstrated different responses of leukocytes, both innate and adaptive immune cells, to ancestral SARS-CoV-2, the Delta variant, and the Omicron variant, indicating their roles in COVID-19 infection and mortality. By studying the correlation changes in leukocyte subtypes between the three types of infection, we can better evaluate the prognosis of COVID-19 patients at admission.

## Data Availability

The raw data supporting the conclusions of this article will be made available by the authors, without undue reservation. Raw and analyzed quantification data have been deposited in the Harvard Dataverse repository (https://dataverse.harvard.edu/dataverse/COVID-19_2025).

## Supplementary material

Supplementary Figure 1 The predicted probability of COVID-19 mortality in relation to the concentrations of leukocytes and the subtypes is stratified by three types of COVID-19 infection: the ancestral strain **(A, D, G, J, M)**, Delta variant **(B, E, H, K, N)**, and Omicron variant **(C, F, I, L, O)**. The gray areas indicate 95% confidence intervals in each panel.
