# Supplementary material for: Different patterns of leukocyte immune responses to infection of ancestral SARS-CoV-2 and its variants
Source: Front Cell Infect Microbiol. 2025 Apr 17;15:1508120. doi: 10.3389/fcimb.2025.1508120 (PMC12043629; doi:10.3389/fcimb.2025.1508120)
Supplement: Supplementary Figure 1 — The predicted probability of COVID-19 mortality in relation to the concentrations of leukocytes and the subtypes is stratified by three types of COVID-19 infection: the ancestral strain (A, D, G, J, M), Delta variant (B, E, H, K, N), and Omicron variant (C, F, I, L, O). The gray areas indicate 95% confidence intervals in each panel. [file Image1.pdf]

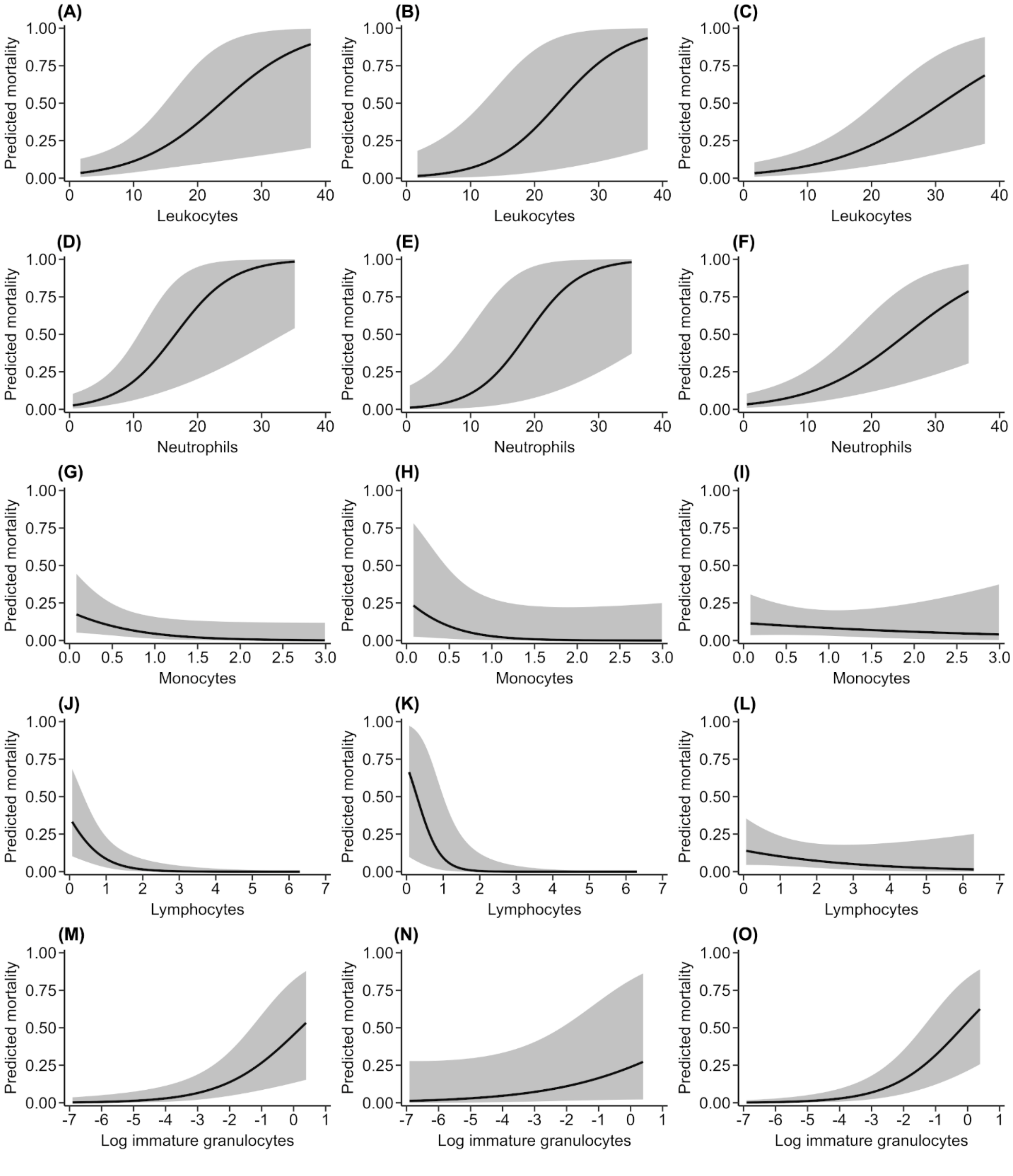

Supplementary Figure 1. The predicted probability of COVID-19 mortality in relation to the concentrations of leukocytes and the subtypes is stratified by three types of COVID-19 infection: the ancestral strain (A, D, G, J, M), Delta variant (B, E, H, K, N), and Omicron variant (C, F, I, L, O). The gray areas indicate 95% confidence intervals in each panel.
